# Unraveling the Role of Lac Insects in Providing Natural Industrial Products

**DOI:** 10.3390/insects13121117

**Published:** 2022-12-05

**Authors:** Nawaz Haider Bashir, Huanhuan Chen, Shahzad Munir, Weiwei Wang, Hang Chen, Yong-Kang Sima, Jiandong An

**Affiliations:** 1 College of Biological Resource and Food Engineering, Qujing Normal University, Qujing 655011, China; 2 Key Laboratory of Insect-Pollinator Biology of Ministry of Agriculture and Rural Affairs, Institute of Agricultural Research, Chinese Academy of Agricultural Sciences, Beijing 100193, China; 3 State Key Laboratory for Conservation and Utilization of Bio-Resources in Yunnan, Yunnan Agricultural University, Kunming 650201, China; 4 Institute of Highland Forest Science, Chinese Academy of Forestry, Kunming 650224, China; 5 Yunnan Academy of Forestry and Grassland Science, Kunming 650201, China

**Keywords:** host plants, interaction, *Kerria*, lac genes, microbial diversity, natural enemies

## Abstract

**Simple Summary:**

Since ancient times, people have used insects, such as scale insects (lac dye, resin, and wax), honey bees (honey and wax), and silkworms (silk), to make natural products in many ways. Lac is the only natural raw material derived from insects, being obtained from several species of the genus *Kerria*. Lac is commercially cultivated in southeastern Asian countries, including China (*Kerria yunnanensis*), India (*K. lacca*), Thailand (*K. chinensis*), and Myanmar (*K. nepalensis*). It is non-toxic, insulating, moisture-resistant, adhesive, tasteless, and has a smooth texture. Lac has been historically employed to finish a small number of products, but it is currently used in various fields. In this review, we mainly focus on the importance of lac genes that are responsible for lac secretion. In addition, information gaps, potential future research directions, and areas that require further research are highlighted.

**Abstract:**

In the current era, products made from organic materials enjoy a privileged position because of their inherent safety. The eco-friendly properties of natural lac resins have increased their demand in many industries. It is secreted by sucking insects (Hemiptera, Kerriidae) and comprises three major components, viz., resin, dye, and wax. Lac insects are generally bivoltine in nature and are distributed in tropical and sub-tropical regions with complex multi-trophic habitats. Because of their sedentary habits, lac insects are more vulnerable to predators, parasitoids, squirrels, and rats, leading to a more than 50% reduction in production yield. To increase lac production, advanced-level molecular research is required to figure out the mechanism behind lac synthesis and secretion to improve lac yield and quality. The present review highlights metamorphosis, sexual dimorphism, multi-trophic habitat, host plants, and natural enemies of lac insects, lac composition, and applications, emphasizing the role of microbes, potential lac genes, and lac synthesis mechanisms in enhancing lac quality and production. The information provided here might be useful for lac researchers and for stakeholders aiming to make their products more eco-friendly.

## 1. Introduction

Lac is a combination of natural resins produced by scale insects [1]. At first, the use of lac was limited to applications such as in decorating wood and as an adhesive for ceramics [2]. It is currently used in a wide range of applications, such as coating pharmaceutical pills, food, musical instruments, material conservation, painting, and electronics insulation, as well as in the military industry [3,4]. Lac has insulation properties, adhesiveness, tastelessness, moisture resistance, and a smooth film texture, and is non-toxic [5,6,7]. Due to these properties, lac is anticipated to be widely used in the future for finishing many industrial products [1,8,9]. Lac production is limited to south, east, and southeast Asian countries such as China, India, Pakistan, Bangladesh, Indonesia, Vietnam, Laos, and Myanmar [10,11].

**Figure 1:**
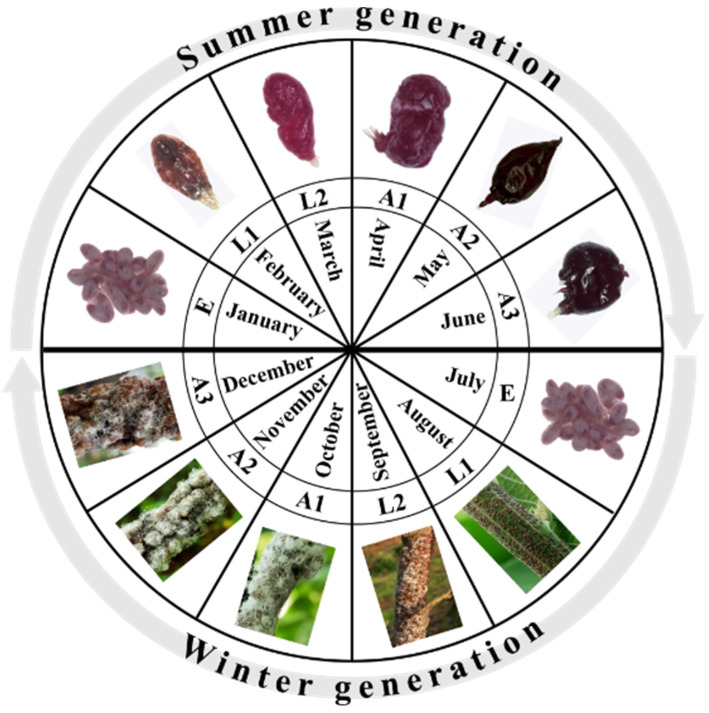
Bivoltine life cycle of female *Kerria lacca*: E represents eggs, L1 first-instar larvae, L2 second-instar larvae, A1 early-adult, A2 mid-adult, and A3 late-adult stage; the summer generation of lac insect shows different forms of lac, and the winter generation shows the attachments via their lac test to the host plant [18,19].

Lac insects are members of the family Kerriidae (Hemiptera), also known as scale insects, which produce a resinous material that forms a hard scale test over their bodies [12,13]. This family is described based on the sclerotized characteristics of an adult female [14]. These are sucking insects that live and feed on various plants [15]. These insects prefer warm climates and are distributed in tropical and sub-tropical regions [10]. Lac insects have sexual dimorphism; females and males have different life cycles and morphology (Table 1) [4]. The male is holometabolous, while the female shows paurometabolous metamorphosis. The adult male is mobile and has legs, well-developed antennae, simple eyes, and no mouthparts [16]. The adult female is immobile and bound in resinous secretion, with a sac-like body appearance, vestigial antennae, and no body segmentation, legs, and eyes [17]. Generally, lac insects are bivoltine, having winter and summer generations every year, from July to December and January to June, respectively (Figure 1) [18]. Female larvae and males can produce a small amount of lac. The female can produce and secrete reasonable amounts of lac at the adult stage [2].

The family Kerriidae comprises 9 known genera and 101 species [13,15]. The members of the genus *Kerria* can produce true lac [18]. Presently, the genus *Kerria* contains 29 species worldwide [13], among which the highest number of species is distributed in India (24), followed by China (11) and Thailand (7), with a few species also being reported in Pakistan, Myanmar, and Vietnam [15]. Some species in this genus, such as *Kerria chinensis*, *K. yunnanesis*, *K. lacca*, *K. ruralis*, *K. sindica*, *K. nepalensis*, and *K. pusana*, can produce industrial lac [4].

Lac insects would not exist without the support of host plants [20], and up to now, more than 400 lac host plants have been recorded [21]. These insects fulfill their nourishment by sucking the phloem sap of host plants [22]. Lac insects are very particular in their selection of host trees. They remain attached to the host tree throughout their life, except the crawler and male adult [10].

Polyterpene esters and hydroxyl fatty acids are both parts of lactones and lactides, which are major components of lac resin [5]. The biochemical pathways and molecular mechanisms for lac production and secretion are not yet clear. However, Wang et al. [4] identified 28 candidate genes involved in lac production and secretion, including genes involved in fatty acid synthesis (which may produce hydroxyl fatty acids), terpenoid biosynthesis (which may be involved in the production of sesquiterpene acids), and UDP-driving glycosylation (which indirectly or directly takes part in the accumulation of activated sugars). Another study identified more than ten genes that may be involved in the biosynthesis pathway of the lac dye [23].

Based on overall studies reported about lac insects and their industrial use, the present review is intended to highlight the available studies on the trophic levels, host plants, natural enemies, molecular investigations, and native microbial diversity of lac, which could open new doors for future advancements in lac production. We focus on highlighting the lac genes that are responsible for lac secretion and production. Moreover, knowledge gaps, future research directions, and areas that need in-depth research are underlined.

## 2. Lac Insect Interaction

**Figure 2:**
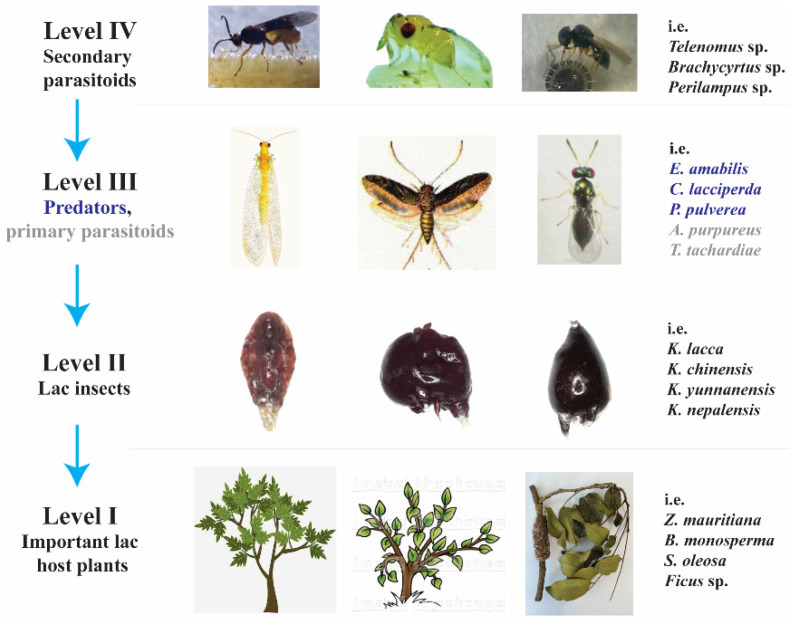
Energy pyramid from levels I to IV of lac insect; source: Bashir et al. and Mishra and Kumar [19,26,27].

The habitat of the lac insect is intricate, with a multi-trophic web of fauna and flora. It includes a rich biodiversity of lac host plants, pests of host plants, lac insects, lac insect predators, microbes, harmful parasites, and beneficial parasites [21]. Lac insects are associated with more than 30 primary parasitoids, 20 lac predators, and 40 secondary parasitoids [24]. Lac host plants represent the first tropic level, while lac insects and various pests of host plants comprise the second tropic level. Predators with primary parasitoids constitute the third tropic level, and secondary parasitoids (parasitoids of lac insect predators) make up the fourth tropic level (Figure 2) [25].

## 3. Lac Composition and Their Industrial Application

**Figure 3:**
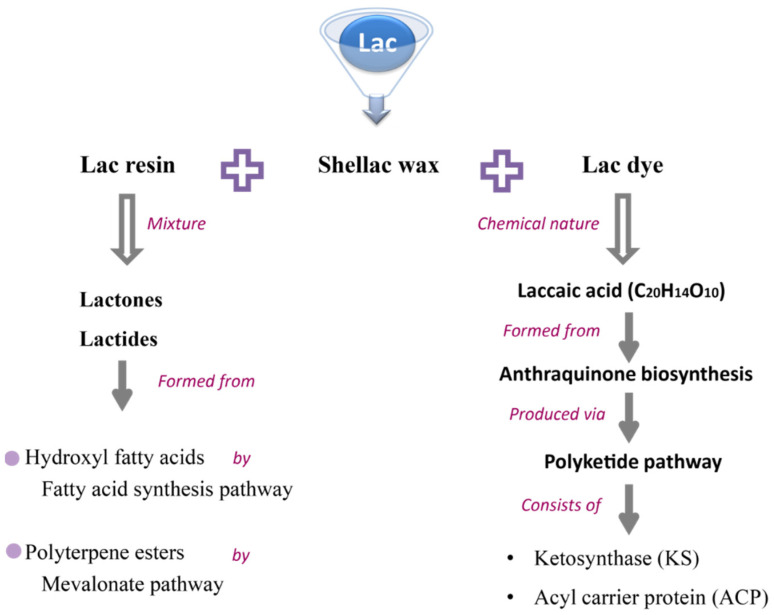
A diagram showing potential biosynthetic pathways for lac resin and lac dye production in lac insects.

Lac is an insect-secreted resinous substance of animal origin [28]. The composition of lac depends on the insect’s species, environmental conditions, and host tree [4]. Generally, lac has three main components, viz., dyes, resins, and shellac wax [29]. Lac resins are mixtures of lactides and lactones, which are formed from polyterpene esters and hydroxyl fatty acids (Figure 3) [5,7]. Lac dye is composed of an anthraquinone derivative, while shellac wax is a mixture of higher alcohol esters, hydrocarbons, and acids. In addition, it has some other chemicals that are used for industrial purposes. Nowadays, lac is considered more valuable as it is biodegradable and provides an opportunity for landless farmers to earn money [30].

**Figure 4:**
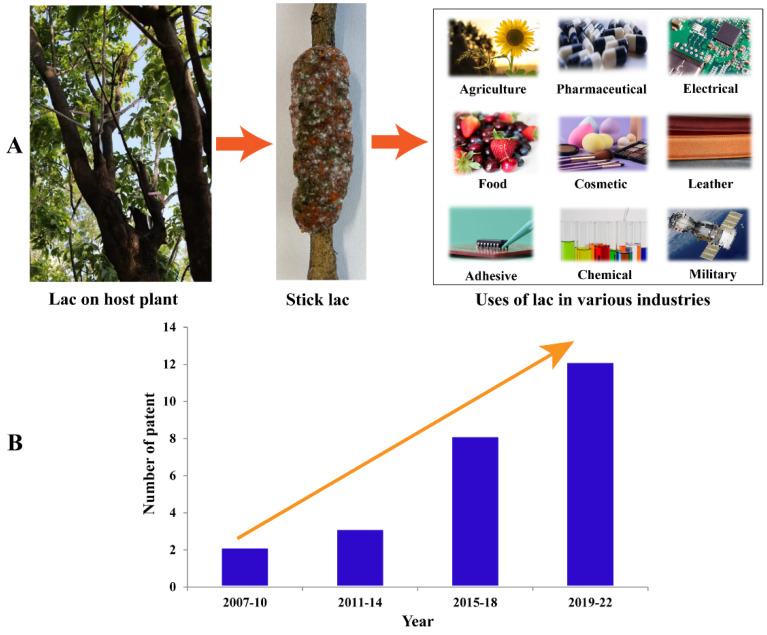
Applications of natural lac products: (**A**) uses in industries, and (**B**) patents on the use of shellac in the last few years.

Lac resins are used in the following industries: the food industry (food-grade ink, fruit coating, non-toxic food packaging printing, chocolates, aluminum foils, and coffee beans); the pharmaceutical industry (coating of tablets and medicines, microencapsulation of vitamins, and marking ink for capsules and tablets); the cosmetic industry (microencapsulated perfumes, bangles, hair spray, lacquer, eye shadows, mascara, lipstick, and nail polish); the electrical industry (green electronics, coating of isolators, PCBs and spark plugs, coating of electric motors and transformers, electrical lamps, transistors, shellac bond powder, air-drying, baking-type, and anti-tracking insulating varnishes); the leather industry (topdressing material, dying and lacquering, leather shoe polishing cream, adhering and coating of leather with plastic and metallic foils); the adhesive industry (optical and gasket cement, sealing wax, fixing steam and water pipes, adhesives for chips and solar cells, and hot melt and polymer adhesives); the varnish, printing, and agriculture industries (floor and furniture polish, sealers, paints for wood, water-soluble lac for earthenware, agent for printing ink (flexible and fast-drying), metal and dry lacquering of wood, heat and waterproof varnish, spray-able varnish, lac-coated urea, and weedicide, nematicidal activity, synthesis of sex pheromones and PGRS, for mosquito control, slow-release fertilizer, and pesticide); and miscellaneous uses (polishing stone, protective lacquer for silvering mirrors, preparation of buttons and toys, high-efficiency igniter-cum-fuel, jewelry, diamond and crystal cutting, proofing, and stiffening in hat making) (Figure 4A) [1,9,31].

In addition to these, byproducts of lac wax and dye are widely used. Lac wax is used in fruit coating, bottle sealer, chalk, electrical potting compounds, lipstick, shoe automobiles, and floor polishes. Lac dye is used in pill coatings, food coatings, soft drink formulations, confectionery, chocolate coating, and the dyeing of wool and silk [32]. In recent years, most lac-related patents have been registered in the food industry, followed by the pharmaceutical, electrical, adhesive, varnish, and ink industries [9,33,34,35,36,37,38,39,40,41,42,43] (Figure 4B).

## 4. Important Lac Genes Involved in Production

Lac resins are mixtures of lactides and lactones, which are formed from polyterpene esters and hydroxyl fatty acids [5]. In the cytoplasm, polyterpene esters are formed through the MVA (mevalonate pathway) [44,45], in which acetyl-CoA is transformed into mevalonic acids by hydroxymethylglutaryl-CoA synthase and reductase. Finally, mevalonic acids are converted to sesquiterpene acids by terpene synthases (TPSs), and sesquiterpene acids are converted into sesquiterpene acid esters [46]. The fatty acid synthesis pathway is responsible for the production of hydroxyl fatty acids by fatty acid synthetases (FASs), elongases of very-long-chain fatty acids (ELO), and fatty acid desaturases (FAD) (Figure 3).

Wang et al. [4] identified 28 candidate genes based on the correlation between gene expression levels and lac accumulation, of which 19 genes are involved in fatty acid synthesis, 4 genes are related to terpenoid biosynthesis, and 5 genes are found in UDP-driving glycosylation. By comparing the expression levels of lac synthesis active and non-active stages in *Kerria lacca*, it was revealed that two genes, acyl-CoA delta desaturase and decaprenyl diphosphate synthase, have an important role in the biosynthesis of lac resin [47]. Shamim et al. [23] selected a few genes that may be involved in the biosynthesis of lac dye, including acyltransferase, enoyl reductase, PKS enzyme partial sequences, 3-oxoacyl-(ACP) synthase II, acetyl CoA acetyl transferase, oxido-reductase, [ACP] S-malonyl transferase, methyl transferase, and oxido-reductase, β subunit. According to Shamim et al. [48], there are two genes (prenyltransferase and cytochrome P450) involved in the multi-step lac resin production pathway.

Bashir and co-workers [19] studied variation in molecular markers during lac secretion minimum and maximum stages of *K. lacca* and found 47 candidate lac-related genes, of which 30 were associated with 114 molecular markers (40 InDels and 74 SNPs). Among these genes, 20 are associated with both InDels and SNPs, with 7 and 13 variants being specifically related to InDels and SNPs, respectively. Finally, based on molecular markers, 23 candidate genes were found to be potentially involved in lac production and secretion, including terpenoid backbone biosynthesis, fatty acid biosynthesis, and fatty acid elongation and metabolism.

**Figure 5:**
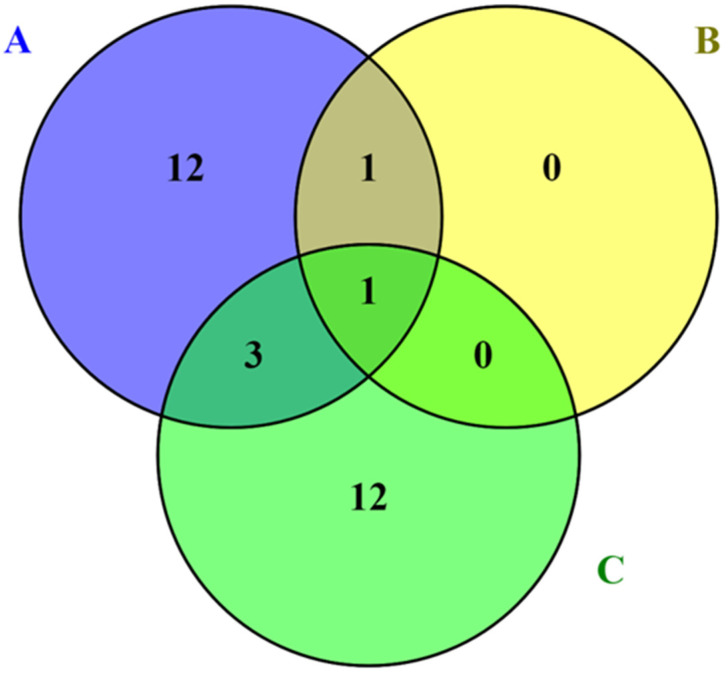
Unique and common genes involved in lac resin production and secretion: (**A**) Bashir et al. [19], (**B**) Kandasamy et al. [47], and (**C**) Wang et al. [4].

Recent research on the genes involved in lac production and secretion found that one gene (decaprenyl-diphosphate synthase) is common in all three studies (ABC), one gene (acyl-CoA delta desaturase) is common in A and B, and three genes (acyl-CoA dehydrogenase, fatty acid synthase, and acyl-CoA synthetase) are common in A and C (Figure 5). Decaprenyl diphosphate synthase is a long-chain polyprenyl diphosphate synthase of the family of prenyltransferases that has important physiological roles in organisms [49,50,51]. Aphids possess decaprenyl diphosphate synthase, a type of isopentenyl diphosphate synthase [52]. Isopentenyl diphosphate synthases convert these precursors to farnesyl diphosphate, and through terpene synthases, farnesyl diphosphates are converted to sesquiterpene [47,53]. Acyl-CoA desaturase, the second most common gene found in both investigations (A and B), is present in animals, bacteria, and fungi [54]. Insect acyl-CoA desaturase has been extensively researched in *Drosophila melanogaster* [55], *Tribolium castaneum* [56], *Acheta domesticus* [57], *Adelphocoris suturalis* [58], and *Nilaparvata lugens* [59] and has been shown to be important in fatty acid biosynthesis [60], cold tolerance [61], eating behavior [62], perception of semiochemical sensing [63], and larval development [64].

## 5. Deciphering the Transcriptomic of Lac Secretion Mechanism

Transcriptomic studies have provided the knowledge needed to understand genome expression [65] and help us recognize the structure of genes, gene regulation, gene expression, and genome dynamics in an organism [66]. Many studies have dealt with transcriptomic analysis and revealed various mechanisms, such as induced defense responses against whitefly (*Bemisia tabaci*) and aphid (*Aphis gossypii*) [67], embryogenesis in brown planthopper (*Nilaparvata lugens*) [68], mechanisms involved in the maternal effect on egg diapause against locusts (*Locusta migratoria*) [69] and silkworm (*Bombyx mori*) [70], mechanisms of stress responses against cutworm (*Spodoptera litura*) [71], and mechanisms of heat tolerance in snout moth (*Glyphodes pyloalis*) [72].

**Figure 6:**
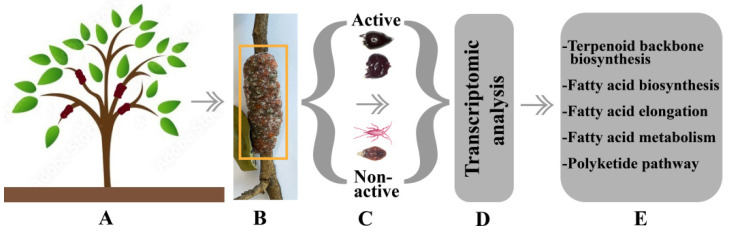
A sketch exploring the mechanism involved in lac secretion: (**A**) host plant, (**B**) stick lac, including lac and lac insect stages, (**C**) female adult stages represent active lac secretion stages, and larval and male stages represent non-active lac secretion stages, (**D**) investigation of mechanisms based on expression levels, and (**E**) different lac production pathways as described in the literature.

Previous studies showed that fatty acid synthesis, terpenoid biosynthesis, UDP-driving glycosylation, and polyketide pathways are involved in the synthesis of shellac. An annotation of *Kerria chinensis* elucidated almost 150 genes found in terpenoid backbone biosynthesis, about 15 of which are involved in sesquiterpenoid biosynthesis, and more than 700 genes are known to be fatty-acid-related pathways [4]. Several studies have also provided supposed pathways and candidate genes that may be involved in the synthesis of lac resin and lac dye [23,48]. Nevertheless, the evidence-based molecular mechanisms and metabolic pathways of lac synthesis are still unknown in lac insects. Omics investigations are required to elucidate the mechanisms and pathways by comparison between lac secretion active (early, mid, and late female adult) and non-active (larval and adult male) life stages of lac insects (Figure 6).

## 6. Role of Native Microbial Diversity of Lac Insects

The native microbial diversity of any host plant plays a significant role in its interaction with the environment and helps in its resistance against invading enemies in the form of other insects or pathogens. We suggested above that most of the lac genes and expression of lac genes in insects are important for production of lac; however, the role of microbes present in lac insects should not be denied a role in lac production. The roles of many microbes have not yet been explored, but these beneficial microbes have hidden arsenals that could be studied in more detail in future research. Bacterial study revealed that early stages of *Kerria lacca* contained *Mucilaginibacter* and *Wolbachia*, whereas adult female contributed genera *Pantoea* and *Wolbachia* [73]. Other studies also reported a few bacterial genera from lac insects, such as *Bacillus*, *Micrococcus*, *Wolbachia*, *Arthrobacter*, *Curtobacterium*, and *Clostridium* [74,75,76]. Yeast-like fungal symbionts are also present in lac insects [77].

Many types of symbiosis have been identified in different insect orders, such as Hemiptera, Diptera, Lepidoptera, and Isoptera. Insects harbor microbes in the digestive tract, on the integuments, and in some unique structures within their bodies [78,79]. The roles of microbes in insects include protection, resistance, digestion, nutrition, production, and communication [73,80]. Symbiosis with bacterial communities is obligatory in insects [81] whose diet is imbalanced, such as sap-sucking insects (phloem sap), termites (wood), and mosquitoes (blood feeder) [77]. Since lac insects are sap feeders [82], it is observed that indigenous microbiota must contribute to the provision of nutrients (amino acids and vitamins that are not obtained from phloem) and other physiological activities [73,75]. As mentioned earlier, a few bacteria that may be beneficial for lac production have been reported in lac insects [77]. However, the significance of the presence of these candidate isolates is still unknown [75].

## 7. Important Host Plants for Lac

**Figure 7:**
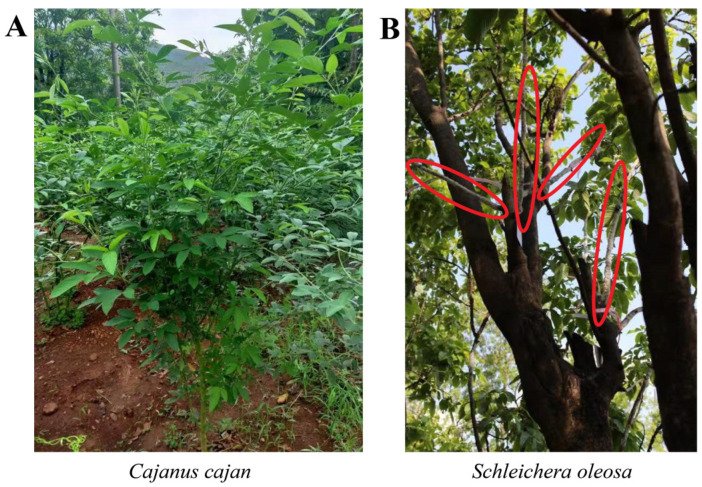
Host plants of lac insects: (**A**) *Cajanus cajan* and (**B**) *Schleichera oleosa* with lac encrustation (red area), Yuanjiang Experimental Station, Institute of Highland Forest Science, Chinese Academy of Forestry, Yunnan Province, China.

Lac insects depend on plants as hosts for their food requirements. Firstly, 4 species are known as host plants of lac insects [83]; recently, more than 400 plant species have been reported worldwide [11,84]. These plants are divided into three types, common, occasional, and rare, as per their suitability and distribution [85]. Occasional and rare host plants do not sustain lac crops; so, these plants are important for preserving some rare lac insect species only [27]. Three species of common host plants, *Schleichera oleosa* (nagoli or kusumr) (Figure 7), *Butea monosperma* (dhak or palas), and *Ziziphus mauritiana* (ber), are considered important for lac production. The quick-growing lac hosts are *Cajanus cajan* (Figure 7), *Flemingia semialata*, *F. macrophylla*, *Acacia nilotica*, *A. catechu*, *A. auriculiformis*, and *Ziziphus mauritiana*, and they are capable of sustaining a lac crop over one generation (Table 2) [27].

Indian wild ber serves as a host plant for lac insects in different agro-climatic zones of Punjab, India [84]. In India, peepal (*Ficus religiosa*), ber (*Ziziphus mauritiana*), litchi (*Litchi chinensis*), kikar (*Acacia nilotica*), and sirin (*Albizia* spp.) are known as major host plants of lac insects [84,88,89], whereas *Acacia nilotica*, *A. catechu*, *Samanea saman*, *Butea monosperma*, and *Ziziphus mauritiana* are common lac host plants in Bangladesh [90]. The various lac host plants in China are *Dalbergia assamica*, *D. szemaoensis*, *D. obtusifolia*, *Ficus altissima*, *F. racemosa*, and *Pueraria tonkinensis* [3,91].

Seven species, including *K. chinensis*, *K. fici*, *K. lacca*, *K. pusana*, *K. maduraiensis*, *K. destructor*, and *K. canalis*, are known to feed on the rain tree host plant *Samanea saman* with slight to severe levels of lac encrustation [13]. Nevertheless, *K. destructor* has been reported to kill large rain trees [28] via repeated generations, aggressive multiplication, and improper management (pruning, watering, fertilizing, weed prevention, disease control, and pest management). Other lac insects form severe encrustation on the branches and twigs without showing any symptoms of dieback. These species might cause chronic damage to the host plant if it is not properly managed for a long time.

## 8. Natural Enemies of Lac Insects and Their Management

Lac crop losses are higher than agricultural crop losses [92]. Lac pests destroy around two-thirds of the feasible lac crop, leaving behind only one-third for the cultivation to harvest. Lac insects live on host plants; being of a sedentary nature, they are more susceptible to being attacked by a number of predators and parasitoids [27,93]. Primary (35 species) and secondary (45 species) parasitoids have been reported to belong to the superfamily Chalcidoidea [24]. Among these, more harmful parasitoids of lac insects are *Aprostocetus* (*Tetrastichus*) *purpureus* (Cameron), *Tachardiaephagus tachardiae* (Howard), and *Parechthrodryinus clavicornis* (Cameron) [94]. However, 20 predators of lac insects have been recorded, including the major predators *Eublemma amabilis* Moore (white moth), *Pseudohypatopa pulverea* Meyr (black moth), and *Chrysopa* sp. (green lace wing) [1,16], which are responsible for 35–40% of lac crop losses as reported by earlier work [94,95]. In the winter season, the sporadic pests *Chrysopa lacciperda* and *C. madestes* are most prevalent and cause significant mortality in lac insects [20,92]. During the rainy season, Sharma, et al. [96] observed 26% and 18% parasitization in rangeeni and kusmi strains, respectively. They observed up to nine and six parasitoids in a single cell of rangeeni and kusmi strains, respectively. Both squirrels and rats can cause serious damage, decreasing the amount of brood sticks to up to 50%. Fungi and bacteria cause damage to the lac crop by affecting lac insects at different stages [94].

The best way to protect crops from pests is to follow preventive measures such as (a) selecting mature, healthy, and brood lac free of parasitoids and predators for crop inoculation (b) after 2–3 weeks of inoculation, removing sticks with no brood lac [94] (c) scrapping excessive stick lac, and (d) processing as early as possible after harvesting the crop [27]. Early inoculation (10–15 days), trap cropping, and intercropping (okra and cotton) techniques can be used as cultural control. In mechanical control, synthetic net container bags (sixty mesh) may be used for inoculation of brood lac [20]. Nymphs will crawl out from minute pores and settle on the twigs of plants, while emerging adult predator moths became entrapped within the net. Effective control of *Pseudohypatopa pulverea* and *Eublemma amabilis* can be achieved by the use of the biopesticide *Bacillus thuringiensis* Berliner in the field. Two ant predators (*Solenopsis geminata* and *Camponotus compressus*) have been evaluated for control of lac predators via attacking the pupae and larvae of *P. pulverea* and *E. amabilis*. Several egg parasitoids have been used for the management of lac predators such as *Telenomus remus*, *Trichogrammatoidea bactrae*, *Trichogramma brasiliensis*, *T. pretiosum*, and *T. chilonis*. Pests can be controlled by applying systemic insecticides that are safe for lac nymphs and effective against predators. A combination of the above components may reduce the pest population and increase lac yield [16,20,27].

## 9. Conclusions and Future Research Directions

In view of the biosafety of natural products, this review highlights detailed information, including the life stages, distribution, and interaction of lac insects; their applications and composition and the lac genes involved in lac synthesis; and microbial diversity, host plants, and natural enemies of lac insects. The factors contributing to yield reduction during lac crop cultivation are highlighted, as well as how they might be improved. Lac insects are attacked by vertebrate and invertebrate pests, resulting in yield losses of up to 50%. This loss can be reduced through timely and effective management. Earlier investigations identified a few genes that may be responsible for lac production, and at least four to five common genes that need to be verified through gene knockout or gene silencing techniques. Until now, there has been no information about indigenous associations involved in lac production. Further investigations are required to determine the role of native microbes in the female lac insect, as this is one of the most important factors in lac yield because commercial lac is obtained solely from female lac insects. In addition, the response of native microbes already present in lac insects and the lac resins on trees during the introduction of identified candidate microbes from different parts of lac insects could be investigated. Advanced omics research (transcriptomics, genomics, metabolomics, and proteomics) could help us to identify the mechanisms and pathways of lac synthesis, which would enhance lac yield.

## Figures and Tables

**Table 1:** Main difference between adult female and male of lac insects.

Character	Female	Male
Metamorphosis	paurometabolous	holometabolous
Body	larger and degenerated	smaller and not degenerated as female
Head, thorax, and abdomen	indistinct	distinct
Eyes	absent	present
Antennae	vestigial	well-develop
Legs	absent	present
Wings	absent	present/absent
Mouthparts	present	absent
Movement	enclosed in lac secretion and neotenic	not enclosed in lac secretion and move freely

**Table 2:** Host plants of lac-producing species of the genus *Kerria*.

Species	Host plant	References
*K. chinensis*	*Cajanus indicus*, *Schleichera* sp., and some other host plants	[86]
*K. lacca*	*Ziziphus mauritiana*, *Butea monosperma*, *Ficus religiosa*, *F. indica*, and some other host plants	[15,87]
*K. nepalensis*	*Litchi chinensis*, *Dalbergia cochinchinensis*, *Ficus* sp.	[26]
*K. pusana*	*Ziziphus jujube*, *Butea frondosa*, *B. monosperma*, *Schleichera oleosa*, *Samanea saman*, *Acacia montana*	[3,15,86,87]
*K. ruralis*	*Mallotus philippinensis*, *Pueraria tonkinensis*	[3,86]
*K. sindica*	*Acacia arabica*, *Albizia lebbeck*, *Ziziphus jujube*, *Ficus racemosa*, *F. glabella*	[86,87]
*K. yunnanensis*	*Dalbergia obtusifolia*, *D. assamica*, *D. mimosoides*, *Schleichera oleosa*, *Ficus microcarpa*	[86,87]

## Data Availability

All data presented in this study are available in the article.
